# Microbial Communities and Flavor Compounds during the Fermentation of Traditional Hong Qu Glutinous Rice Wine

**DOI:** 10.3390/foods11081097

**Published:** 2022-04-11

**Authors:** Anqi Liu, Xu Yang, Quanyou Guo, Baoguo Li, Yao Zheng, Yuzhuo Shi, Lin Zhu

**Affiliations:** 1 East China Sea Fishery Research Institute, Chinese Academy of Fishery Sciences, Shanghai 200090, China; laqshlg@163.com (A.L.); yangxu@ecsf.ac.cn (X.Y.); zhengyao@ecsf.ac.cn (Y.Z.); syzxst2021@163.com (Y.S.); zhulinlynne@hotmail.com (L.Z.); 2 School of Health Science and Engineering, University of Shanghai for Science and Technology, Shanghai 200093, China; lbaoguo@126.com

**Keywords:** Hong Qu glutinous rice wine, microbial diversity, flavor compounds, dynamic change, correlation analysis

## Abstract

As a traditional Chinese rice wine, Hong Qu glutinous rice wine (HQW) is popular among consumers due to its unique flavor. However, its quality changes during fermentation, and the potential relationships between flavor and microbes have not been systematically researched. In this work, physicochemical properties (pH, total sugar, alcohol, amino acid nitrogen), flavor compounds (organic acids, free amino acids, and volatile compounds), and microbial communities were investigated. The results revealed that *Pantoea*, *Lactiplantibacillus*, *Lactobacillus*, *Leuconostoc*, and *Weissella* predominated the bacterial genera, and *Monascus* was the predominant fungal genus. Organic acids, free amino acids, and key volatile compounds (esters and alcohols) significantly increased during fermentation. The correlations analysis showed that *Lactiplantibacillus* was closely associated with flavor compounds formation. This study deepens our understanding of the roles of microorganisms in flavor formation on traditional HQW fermentation.

## 1. Introduction

Hong Qu glutinous rice wine (HQW), a famous traditional Chinese rice wine invented in the Song Dynasty, has a red color, a special flavor, and a sourish taste, which is believed to improve digestion and blood circulation. Traditional HQW is brewed by open fermentation in pottery jars, and its process includes macerating and steaming rice, saccharification, fermentation, pressing, and storage. This traditional brewing process cannot meet the demand of consumers, so many companies expand the production of HQW through industrialized methods. However, there is a significant difference in flavor and quality between industrial and traditional HQW. Thus, it is necessary to conduct a comprehensive analysis of the flavor changes of traditional HQW during the fermentation process to provide a scientific reference for industrial production.

The complex flavor of HQW consists mainly of organic acids, free amino acids, and volatile flavor compounds, which are obtained from the raw materials and fermentation. More precisely, fermentation increases the concentration of organic acids, free amino acids, and volatile compounds, and is the main contributor to the formation of key flavors in rice wine [1]. During the saccharification stage, filamentous fungi promote the hydrolysis of starch to produce sugar, leading to a more rapid flavor formation in rice wine [2]. Microorganisms use fermentable sugars to produce large amounts of organic acids by carbohydrate metabolism [3]. Organic acids provide the main sour taste to the rice wine and improve the color of rice wine [4]. When some microorganisms undergo autolysis, proteases release to promote the production of free amino acids. The free amino acids contribute to the complex taste of the food including umami, sweet, bitter, and astringency [5]. As precursors to volatile compounds, organic acids and free amino acids contribute to the formation of aromas in rice wine due to the metabolic action of varieties bacteria, fungi, and yeasts [6]. In complex fermentation environments, different microflora may produce different flavors for rice wine. During the fermentation of traditional *Guizhou* black *Huangjiu*, *Gluconobacter*, *Bacillus*, *Streptococcus*, *Lactobacillus*, *Lactococcus*, *Pediococcus*, and *Leuconostoc* have been shown to influence the change in metabolites (esters, alcohols, acids, and alkanes) [7]. During the fermentation of traditional *Wuyi Huangjiu*, *Lactobacillus*, *Pichia mississippiensis*, and *Saccharomyces cerevisiae* were positively correlated with ethyl ester formation, while *Bacillus myloliquefaciens* and *Bacillus subtilis* were negatively correlated with volatile acids and esters [8]. Thus, it is necessary to analyze the flavor compounds and microbial communities of HQW during fermentation, which helps to elucidate which microorganisms play the different roles in the flavor formation of HQW.

As an advanced second-generation sequencing technology, high-throughput sequencing (HTS) overcomes the limitations of traditional molecular methods. Previous studies have reported that HTS could reveal the complex microbial community more precisely and comprehensively [9]. Thus, HTS has been widely used for comprehensive analysis of microorganisms in various fermented foods, such as black glutinous rice wine [10], Chinese rice wine [11], highland barley wine [12], and fermented fish [13]. The detection means for flavor compounds include high-performance liquid chromatography (HPLC), liquid chromatography–mass spectrometry (LC–MS), gas chromatography–mass spectrometry (GC–MS), and gas chromatography–ion mobility spectrometry (GC–IMS). As an emerging technique, GC–IMS has the advantages of a high sensitivity, a low detection limit for the ppbv level, no requirement for the pretreatment of samples, secondary separation, an improved separation effect, three-dimensional visual spectrum formation, and a direct comparison between spectra with plugins. Yang used GC–IMS to analyze volatile flavor compounds across different production stages of fermented soybean whey tofu (FSWT), and found that ketones, alcohols, and esters contribute to the unique flavor of the final FSWT product [14].

This study aims to detect the flavor compounds of HQW and analyze the microbial dynamics including fungal and bacterial communities during fermentation. It is hypothesized that physiochemical properties could reflect the degree of traditional HQW fermentation, and there is a clear link between the microbial community and various flavor compounds. Furthermore, results from the current study would provide new insights for improving the flavor and quality of HQW by screening core microbial communities.

## 2. Materials and Methods

### 2.1. Sample Preparation and Collection

HQW was brewed by traditional brewing methods. An amount of 4 kg of glutinous rice was washed and soaked in water at room temperature (around 15 °C) for 10 h, drained, and then steamed in 100 °C for 30 min. After, the steamed rice was cooled to 35 °C in a ventilated and cool environment. The cooled steamed glutinous rice was mixed with 6 L of cool sterile water and 400 g of Hong Qu fermentation starters in a clay vat and then fermented at 20 °C for 30 days.

At 0, 6, 12, 18, 24, and 30 days of fermentation, 10 g of HQW was randomly sampled for microbial diversity testing. At the same time, 500 mL of fermentation mash was collected for analysis of physicochemical properties, organic acids, free amino acids, and volatile flavor compounds (VFCs).

### 2.2. Physiochemical Properties Determination

The pH of the sample was tested by a pH meter. The alcohol (percentage of alcohol by volume), total sugar, and amino-acid nitrogen were measured by an alcohol meter and titration method according to Chinese national standard GB/T13662-2018.

### 2.3. Total DNA Extraction and PCR Amplification

Total genomic DNA samples were extracted using the OMEGA Soil DNA Kit (D5625-01) (Omega Bio-Tek, Norcross, GA, USA). The quantity and quality of extracted DNAs were measured using a NanoDrop ND-1000 spectrophotometer (Thermo Fisher Scientific, Waltham, MA, USA) and agarose gel electrophoresis, respectively. The V3-V4 region of the bacteria 16S rRNA gene was amplified with primers 338F (5′-ACTCCTACGGGAGGCAGCA-3′) and 806R (5′-GGACTACHVGGGTWTCTAAT-3′), and the ITS1 region of the fungi was amplified with the forward primer ITS1F (5′-CTTGGTCATTTAGAGGAAGTAA-3′) and the reverse primer ITS1R (5′-GCTGCGTTCTTCATCGATGC-3′).

Sample-specific 7-bp barcodes were incorporated into the primers for multiplex sequencing. The PCR components contained 5 μL of buffer (5×), 0.25 μL of Fast pfu DNA Polymerase (5 U/μL), 2 μL (2.5 mM) of dNTPs, 1 μL (10 μM) of each Forward and Reverse primer, 1 μL of DNA Template, and 14.75 μL of ddH_2_O. Thermal cycling consisted of initial denaturation at 98 °C for 5 min, followed by 25 cycles consisting of denaturation at 98 °C for 30 s, annealing at 53 °C for 30 s, and extension at 72 °C for 45 s, with a final extension of 5 min at 72 °C. PCR amplicons were purified with Vazyme VAHTSTM DNA Clean Beads (Vazyme, Nanjing, China) and quantified using the Quant-iT PicoGreen dsDNA Assay Kit (Invitrogen, Carlsbad, CA, USA). After the individual quantification step, amplicons were pooled in equal amounts, and pair-end 2 × 250 bp sequencing was performed using the Illlumina NovaSeq platform with the NovaSeq 6000 SP Reagent Kit at Shanghai Personal Biotechnology Co., Ltd. (Shanghai, China)

### 2.4. Sequence Analysis

Microbiome bioinformatics were performed with QIIME2 2022.2 with slight modification according to the official tutorials (https://docs.qiime2.org/2022.2/tutorials/ (accessed on 5 April 2022)). Briefly, raw sequence data were demultiplexed using the demux plugin following by primers cutting with the cutadapt plugin. Sequences were then quality-filtered, denoised, merged, and the chimera removed using the DADA2 plugin. Nonsingleton amplicon sequence variants (ASVs) were aligned with mafft and used to construct a phylogeny with fasttree2. Taxonomy was assigned to ASVs using the classify-sklearn naїve Bayes taxonomy classifier in the feature-classifier plugin against the SILVA v138.1 (bacteria) and UNITE v8.0 (fungi) Database.

### 2.5. Analysis of VFCs

The VFCs of traditional fermented HQW were detected by using Yang’s modified method [14]. Headspace-solid-phase microextraction (HS-SPME) coupled with gas chromatography–ion mobility spectrometry (GC–IMS) was used to identify VFCs, and an Agilent 490 gas chromatograph (Agilent Technologies, Palo Alto, CA, USA) and IMS instrument (FlavourSpec^®^, Gesellschaft für Analytische Sensorsysteme mbH, Dortmund, Germany) equipped with an automatic sampling device were used to detect the flavor substances in samples collected from different production stages.

A sample of 1 g was added to the 20 mL headspace vial of the automatic sampler. The incubation temperature of the automatic sampler was set at 60 °C, and the incubation time was 10 min. The headspace injection method was selected, with oscillation heating at 500 rpm, an injection temperature of 85 °C, and an injection volume of 100 μL. The sample entered the MXT-WAX capillary column (30 m × 0.53 mm, 1 μm) as a gas. The chromatographic column temperature was set at 60 °C, the carrier gas was N_2_ (purity ≥ 99.999%), and the carrier gas flow rate was programmed as follows: the initial flow rate reached 2.0 mL/min within 2 min; the internal linearity was increased to 10.0 mL/min within 8 min, followed by 100.0 mL/min within 10 min and then to 150.0 mL/min within 10 min; the total running time was 30 min.

### 2.6. Analysis of Free Amino Acids

Free amino acids in standards and samples were detected by high-performance liquid chromatography (HPLC) (Agilent 1260, Agilent Technologies, Palo Alto, CA, USA) equipped with a C18 column (Zorbax Eclipse-AAA, 150 mm × 4.6 mm, 3.5 μm) and a variable-wavelength detector (VWD). Samples of 40 mL were centrifuged at 10,000 rpm for 10 min at 4 °C and were then filtered with a 0.45 μm PTFE membrane. An amount of 1 μL was injected from the filtered samples and the flow rate was maintained at 2.0 mL/min. Two amino acid derivatives were used, including o-phthalaldehyde (OPA) and 9-fluorenylmethyl chloroformate (FMOC). The column temperature was maintained at 40 °C with the detection wavelengths of 262 nm and 338 nm. Eluent A: 0.04 mol/L sodium dihydrogen phosphate (pH is adjusted to 7.8 ± 0.01 by 10% NaOH). Eluent B: methanol, acetonitrile, and water (45:45:10, *v*/*v*/*v*). Amino acid species was identified by retention times and reference standards, and the content was determined by standard curves.

### 2.7. Analysis of Organic Acids

The organic acids of traditional fermented HQW were detected by using Huang’s modified method [8]. Organic acids in standards and samples were detected by HPLC (Agilent 1260, Agilent Technologies, Palo Alto, CA, USA) equipped with a C18 column (Zorbax SB-AQ, 250 mm × 4.6 mm, 5 μm) and a variable-wavelength detector (VWD). Samples of 40 mL were centrifuged at 10,000 rpm for 10 min at 4 °C and were then filtered with a 0.22 μm PTFE membrane. An amount of 1 μL was injected from the filtered samples and the flow rate was maintained at 0.8 mL/min. The column temperature was 25 °C, and the detector wavelength was 210 nm. Only one eluent was used as the mobile phase: eluent A: 0.02 mol/L sodium dihydrogen phosphate and acetonitrile (99:1, *v*/*v*, pH is adjusted to 2 ± 0.01 by 85% H_3_PO_4_).

### 2.8. Statistical Analysis

All the analyses were conducted in triplicate, and then averages and standard deviations were calculated using SPSS 17.0 software (SPSS Inc., Chicago, IL, USA). Analysis of variance (ANOVA) and t-tests were used to evaluate the differences between glutinous rice wines at different fermentation stages. The VFCs were analyzed by principal component analysis (PCA) using SIMCA-14.1 software (UMETRICS, Malmö, Sweden). Correlation coefficients between microbial genera and flavor compounds were calculated using R software (v4.0.4, R Foundation for Statistical Computing, Vienna, Austria), and *p* values were multiply corrected by fdr.

## 3. Results and Discussion

### 3.1. Analysis of the Composition of Microbial Communities

**Figure 1:**
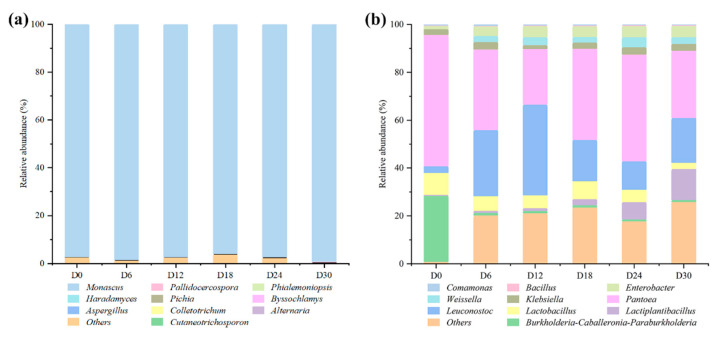
Changes in relative abundances of (**a**) fungi and (**b**) bacteria genera during Hong Qu glutinous rice wine (HQW) fermentation.

The microbial genera dynamics of HQW was analyzed during fermentation. As shown in Figure 1a, *Monascus* was the dominant fungi genus, with a relative abundance greater than 97% during the fermentation, and as high as 99.5% at the end. Previous studies have shown similar results that *Monascus* was detected as the dominant fungi genus in both Wuyi and Gutian HQW [15,16]. *Monascus* is a type of filamentous fungi and was first screened in red mold rice and characterized by van Tieghem (1884). *Monascus* can secrete a variety of hydrolytic enzymes including α-amylase, glucoamylase, lipase, and protease, playing an extremely important role in the glycation phase of the fermentation process [2]. As a characteristic species of East Asia, *Monascus* has received worldwide attention for its abundant beneficial flavor compounds, such as monacolin and pigments [15]. Besides *Monascus*, the relative abundance of the other fungi genera was below 0.1% during the fermentation, such as *Pallidocercospora*, *Phialemoniopsis*, *Haradamyces*, and *Pichia*. The alcohol and low pH in rice wine will inhibit the growth of non-acid and alcohol-tolerant microorganisms [1].

The bacterial genera dynamics during fermentation is shown in Figure 1b. The predominant bacteria include *Pantoea*, *Leuconostoc*, *Lactiplantibacillus*, *Lactobacillus*, and *Weissella*. At the beginning, *Pantoea* was the dominant bacterium with an abundance of 54.8%; then, it significantly decreased to 23.2% on day 12, and finally reached 28.1% after a brief fluctuation. *Pantoea* is derived from HQ, which is a common endophyte found in rice [10]. Meanwhile, *Leuconostoc* increased from an initial abundance of 2.8% to 38.0% on day 12, and then fluctuated to a final abundance of 18.7%. *Lactiplantibacillus* continued to increase from an initial abundance of 0.1% to 12.9%, and the abundance of *Lactobacillus* increased from 0.02% to 2.7% during the fermentation. In recent studies, the genus *Lactobacillus* has been reclassified into 25 genera including *Lactiplantibacillus* and the emended genus *Lactobacillus* [17]. Due to the fact that *Lactobacillus* can tolerate low pH and inhibit the growth of other microorganisms, they are usually detected as the dominant genus in most fermented foods [8]. *Weissella* was not identified at the beginning of the fermentation, and its abundance was 2.5% on day 6, indicating that it might be brought in from the environment. In Liu’s study, *Weissella* was shown to promote the formation of acetic acid during the brewing process of soy sauce [18]. During the fermentation process, the proliferation of lactic acid bacteria (LAB) can produce organic acids and bacteriocins, reducing the diversity of microorganisms and inhibiting the growth of some pathogenic bacteria, which make a great contribution to the flavor formation and food safety of wine [13]. Furthermore, their parthenogenic anaerobic growth characteristics and bacteriocin production make lactic acid bacteria dominant in most fermented foods [3]. In addition, *Klebsiella*, *Enterobacter*, and *Bacillus* were also identified with low abundance during the fermentation of HQW. As a ubiquitous Gram-positive bacteria in the environment, *Bacillus* of rice wine is obtained from fermentation starters or the environment, and contribute to the formation of the rice wine flavor by secreting hydrolytic enzymes [19]. However, the growth of *Bacillus* is inhibited due to the increase in alcohol concentrations during the fermentation of rice wine [20]. *Klebsiella* is a common endophyte found in cereals. Along with *Enterobacter*, they were identified in corn silage, where they can carry out carbohydrate fermentation to promote lactic and acetic acid formation and pH reduction [21]. They were also identified with low abundance during the brewing of some grain wines [22].

### 3.2. Physicochemical Properties and Flavor Compounds

#### 3.2.1. Physicochemical Properties Analysis

The physicochemical properties (pH, total sugars, alcohol, amino acid nitrogen) during HQW fermentation are shown in Table 1. These indicators are typically used to determine the fermentation status of rice wine. After 6 days of fermentation, the pH decreased significantly from an initial 5.83 ± 0.01 to 3.86 ± 0.02 (*p* < 0.05), and stabilized during the subsequent fermentation stages, with a final pH of 3.84 ± 0.01. On the other hand, total sugars significantly increased (*p* < 0.05) on day 6 and then significantly decreased (*p* < 0.05) on day 12, gradually decreasing to a final concentration of 1.56 ± 0.08 g/L. Meanwhile, alcohol and amino acid nitrogen kept increasing during the whole fermentation process, similar to the results of another study on the fermentation of black glutinous rice wine [10]. This indicates that the first 6 days were the saccharification stage of fermentation, in which amylase secreted by some filamentous fungi hydrolyzed the starch in glutinous rice to produce large amounts of glucose [23]. Ethanol, organic acids, and other metabolites such as amino acids were synthesized, and the decrease in pH value was obtained.

#### 3.2.2. Changes in the Free Amino Acid Fraction of HQW during Fermentation

As shown in Table 2, the 17 amino acids were identified during fermentation and classified into umami, sweet, bitter, and astringent amino acids according to their taste presentation characteristics. Among them, Trp, Arg, Ile, Glu, and Pro were the main components of free amino acids. During the fermentation process, free amino acids are produced mainly through the enzymatic degradation of proteins in the raw material by microorganisms secreting proteases, as well as by intracellular proteases after autolysis of microorganisms [5]. The most significant increase in four groups of amino acids was observed on day 12 compared to the other stages, with almost 3 times more than the previous stage. The total concentration of four groups of amino acids all continued to increase during fermentation, while the bitter amino acids were the most abundant. Previous studies have indicated that high temperatures increase the content of bitter amino acids during the fermentation of Chinese rice wine [16]. Six of the eight bitter amino acids are essential to the human diet, except for Arg and His. Free amino acids bring umami, sweet, bitter, and astringent flavors to the food, and are also important nitrogen sources for microbial growth and proliferation and can produce many secondary metabolites such as higher alcohols [24]. In this biotransformation process, there is a corresponding relationship between amino acids and higher alcohols, such as isoleucine to 2-methylbutanol, valine to 2-methylpropanol, and leucine to 3-methylbutanol [25].

#### 3.2.3. Changes in Organic Acids

The acidity of HQW results from organic acids, which affect the organoleptic quality of the wine dramatically, and can inhibit the growth of microbes as well as promote the production of esters. They mainly come from chemical synthesis by microbial metabolism such as alcoholic fermentation, malolactic fermentation, and oxidation of the ethanol [26].

Table 3 shows the changes in six organic acids identified during the fermentation of HQW, including lactic acid, succinic acid, citric acid, malic acid, tartaric acid, and oxalic acid. During the fermentation process, the total concentration of organic acids increased continuously. The most abundant organic acid was lactic acid, followed by succinic acid, citric acid, and malic acid. After 6 days of fermentation, lactic acid increased significantly from an initial level of 27.1 ± 0.6 mg/L to 4027.6 ± 6.5 mg/L (*p* < 0.05), then gradually increasing in the following days to a final concentration of 8073.9 ± 46.9 mg/L, accounting for 55.29% of the total organic acids. Rice and grains are the raw materials for HQW, and the rich carbohydrates make lactic acid the main metabolite of LAB [3]. This also involves malolactic fermentation (MLF) under the action of LAB, and MLF is carried out just after the inoculation with a starter culture, the decarboxylation of malic acid into lactic acid and CO_2_ [27]. Thus, the concentration of lactic acid maintains a continuous increase during the fermentation of traditional rice wine. Tartaric acid had the highest abundance at the beginning of fermentation, increased on day 12, and then gradually decreased to a final concentration of 953.9 ± 14.1 mg/L. The initial concentration of malic acid was lower than that of tartaric acid, and gradually increased after 6 days to a final concentration of 1223.2 ± 38.4 mg/L. As an important substrate for MLF, malic acid is always detected in rice wine with a moderate level [11]. The lowest organic acid content was oxalic acid, which fluctuated and remained below 70 mg/L during fermentation. Generally, organic acids bring different acidity perceptions to form the harmonious sour taste: lactic acid gives a soft sour taste, slightly with frankincense to rice wine, citric acid tastes fresh and cool, succinic acid tastes salty and bitter, malic acid represent a bit of sharp sour, and tartaric acid is bitter and tough sour [12].

#### 3.2.4. Volatile Flavor Compounds Analysis

**Figure 2:**
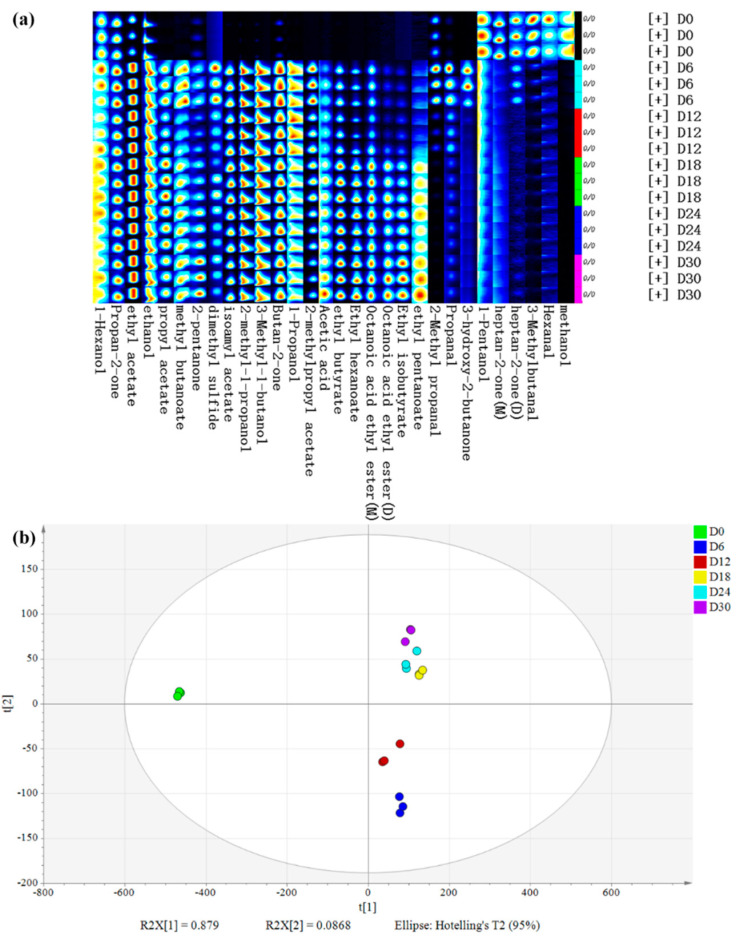
(**a**) Gallery plot and (**b**) PCA of volatile flavor compounds (VFCs) in HQW at different fermentation stages.

The volatile flavor compounds (VFCs) were analyzed during the fermentation of HQW using headspace–gas chromatography–ion mobility spectrometry (HS–GC–IMS). A total of 29 VFCs were identified (Figure 2a), including 11 esters, 7 alcohols, 5 ketones, 4 aldehydes, 1 acid, and 1 other substances. For most VFCs, their concentrations were low at the beginning of fermentation, increased significantly at day 6 and 18, and then after 18 days, the concentration of VFCs became relatively stable. Among them, esters, alcohols, and ketones were the main aromas during HQW fermentation.

Principal component analysis (PCA) was performed to deeply understand the differences in VFCs of samples at different stages of fermentation. PC 1 and PC 2 accounted for 87.9% and 8.68% of the variance in the population, respectively. Figure 2b suggests that the flavors were considerably different between the initial and other fermentation stages, and the difference between day 6 and day 12 decreased, further decreasing from day 18 to day 30. In other words, the differences in VFCs of samples were smaller as the fermentation time increased. The PCA results indicated that 13 compounds were selected because they were more likely to cause differences between samples, including 3 alcohols (3-methyl-1-butanol, 2-methyl-1-propanol and 1-propanol), 7 esters (isoamyl acetate, ethyl acetate, ethyl butyrate, ethyl hexanoate, octanoic acid ethyl ester, 2-methylpropyl acetate, and propanoic acid ethyl ester), and 1 ketone (butan-2-one). This suggests that esters, alcohols, and ketones are more likely to cause differences in volatile flavors in HQW than other volatile compounds, and these compounds were particularly noted in further analyses.

Esters are the most abundant VFCs in HQW, which can provide fruity, sweet, and floral aromas [10]. A total of 11 esters were identified, including ethyl acetate, isoamyl acetate, ethyl hexanoate, octanoic acid ethyl ester, ethyl butyrate, 2-methylpropyl acetate, propanoic acid ethyl ester, ethyl isobutyrate, methyl butanoate, propyl acetate, and ethyl pentanoate, all of which increased significantly on day 6, and then maintained a slight increase or decrease during the remainder of the fermentation phase. Some of esters come from raw materials, but most of esters are produced by the esterase-catalyzed reaction of alcohols and acids produced from glucose and amino acids during microbial metabolism [28]. Generally, the latter is the primary pathway in the formation of esters [29]. Ethyl acetate and isoamyl acetate are the most abundant ester components, which could render good organoleptic characteristics (fruity, sweet, pineapple) to a fermentative process [30]. Ethyl acetate is regarded as the most important ester component influencing the flavor of rice wine, which was also identified as the dominant VFCs in the final stage of highland barley wine brewing, due to its steady formation from the predominant ethanol and acetic acid [12]. All esters increased most significantly on day 6 than in the other brewing stages. During the final brewing stage (12 to 30 days), the ester components whose level increased mostly were ethyl hexanoate, ethyl butyrate, and octanoic acid ethyl ester. Ethyl hexanoate had a fruity aroma, while ethyl butyrate and octanoic acid ethyl ester brought apple-like and pineapple flavors.

Alcohols are the major contributors for the flavor and aroma of HQW. During fermentation, seven alcohols were identified, including: 3-methyl-1-butanol, ethanol, 2-methyl-1-propanol, 1-propanol,1-hexanol, 1-pentanol, and methanol, in which ethanol, 3-methyl-1 butanol, and 2-methyl-1-propanol accounted for more than 95% of the total alcohols. The concentrations of these three alcohols increased significantly on day 6, then remained stable. Alcohols are produced through the metabolism of sugars and the decarboxylation and dehydrogenation of amino acids [6]. In Yang’s study, 3-methyl-1-butanol and 2-methyl-1-propanol accounted for more than 60% of the total alcohols during the fermentation of Hong Qu Huangjiu [31]. Higher alcohols contain more than two carbons and have a higher boiling point, higher cetane number, and higher energy density than those of ethanol [32]. Higher alcohols can usually bring specific aromas to wines and enhance their aroma complexity [10]. For example, 3-methyl-1-butanol is an additive with a banana flavor, which can improve the taste of wine by reducing the bitter-tasting amino acids (leucine) [33]. 1-hexanol presents herbaceous, woody, and sweet aromas, and 1-pentanol and 2-methyl-1-propanol contribute a light wine aroma to rice wine [34].

During fermentation, the concentration of all five identified ketones tended to increase, including: 3-hydroxy-2-butanone, heptan-2-one, 2-pentanone, butan-2-one, and propan-2-one. Among them, the concentrations of butan-2-one, 2-pentanone, 3-hydroxy-2-butanone, and propan-2-one increased significantly on day 6 and then remained stable, except 3-hydroxy-2-butanone, which decreased significantly on day 12. Meanwhile, heptan-2-one decreased significantly on day 6 and then remained stable. Ketones can also bring a specific aroma to the wine; for example, 3-hydroxy-2 butanone presents a milk-like aroma and heptan-2-one presents a special floral aroma [35]. In addition, four aldehydes were identified during fermentation. They are mainly derived from the oxidation and degradation of lipids and have great influence on the flavor of wine due to their fruity, nutty aroma and low odor threshold [36]. During fermentation, 3-methylbutanal and hexanal continued to decrease to a very low final concentration. 3-methylbutanal is naturally present in lemon essential oil and has an apple and peach flavor when highly diluted [37]. The levels of 2-methyl propanal and propanal were highest on day 6, and they presented a pungent odor and were mainly used in the configuration of fruit flavors [32]. During fermentation, only one volatile acid (acetic acid) was identified. Acetic acid is produced during alcoholic and lactic fermentation and presents a pungent odor, and it is an important precursor to ethyl acetate [10].

### 3.3. Correlation Analysis of Microbial Communities and Flavor Compounds

**Figure 3:**
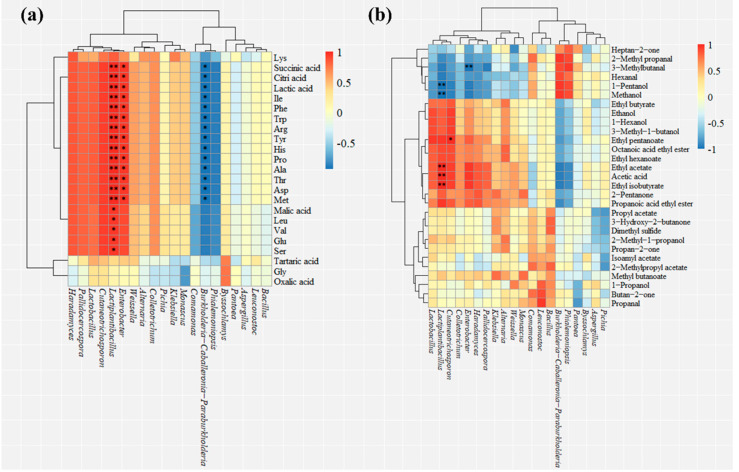
Correlation between the relative abundance of microbial genera and flavor compounds. The Spearman correlation coefficient reflects the correlation between the top 10 dominant bacterial/fungal species with (**a**) free amino acids and organic acids and (**b**) VFCs during the fermentation of HQW. fdr was used to correct the correlation *p*-values, “**” represents significance at *p* < 0.01, “*” represents significance at *p* < 0.05.

The top 10 abundant fungi and bacterial genera were selected to analyze their correlations with flavor compounds by Spearman’s correlation coefficient. To reduce the mistakes due to the increasing number of correlation analyses, correlation *p*-values were calculated and multi-corrected. As shown in Figure 3, each genus had complex correlations with flavor compounds, and significant correlations were marked.

As shown in Figure 3a, *Lactiplantibacillus*, *Lactobacillus*, *Leuconostoc*, *Klebsiella*, *Weissella*, *Enterobacter*, *Bacillus*, *Cutaneotrichosporon*, *Alternaria*, *Colletotrichum*, *Byssochlamys*, *Pichia*, *Haradamyces*, *Pallidocercospora*, and *Monascus* had positive correlations with the formation of most organic acids and free amino acids. Among them, *Lactiplantibacillus* had significant positive correlations with lactic acid, citric acid, succinic acid (*p* < 0.01), and malic acid (*p* < 0.05). During the fermentation of traditional rice wine, the dominant lactic acid bacteria (LAB) produce most of the organic acids due to multiple carbohydrate metabolic pathways. Lactic acid is the main metabolite of LAB in both homo- and hetero-lactic fermentation [3]. In homolactic fermentation, glucose is converted to pyruvate via the EMP pathway, and to lactic acid catalyzed by lactate dehydrogenase [38]. In heterolactic fermentation, the EMP pathway produces pyruvate, and which is metabolized to ethanol and acetic acid by pyruvate formate lyase and pyruvate oxidase [39]. In the carbohydrate metabolism of LAB, pyruvate plays a central intermediate in the branch of the metabolic pathway. *Lactiplantibacillus* promotes the formation of pyruvate to secure the output of metabolic downstream products through the EMP pathway [40]. Thus, *Lactiplantibacillus* promotes the production of citric acid, succinic acid, and malic acid through the tricarboxylic acid cycle (TCA) [41]. *Enterobacter* had significant positive correlations with lactic acid, citric acid, and succinic acid (*p* < 0.05), while *Burkholderia-Caballeronia-Paraburkholderia* was negatively correlated with them (*p* < 0.05). As parthenogenic anaerobic bacteria, *Enterobacter* ferments sugar to produce various organic acids, including lactic acids, succinic acids, acetic acids, and formic acids [21].

Free amino acids are generated through proteolysis of the raw material by proteases from endogenous enzymes or microorganisms. The dynamics of the microbial genera leads to changes in the free amino acid components. As shown in Figure 3a, 16 microbial genera are potentially related to the formation of free amino acids. *Lactiplantibacillus* had significant positive correlations with Ile, Phe, Trp, Arg, Tyr, His, Asp, Thr, Met, Ala, and Pro (*p* < 0.01), and Leu, Val, Glu, and Ser (*p* < 0.05). During food fermentation, the glutaminase activity of LAB promotes the accumulation of glutamate (Glu), which contributes to the freshness and umami of fermented foods [42]. When LAB apoptosis occurs, various free amino acids are released from the cells [5]. Previous studies have described that the total bitter amino acid and total umami amino acid content of HQW were strongly and positively correlated with *Lactobacillus* [16]. Shang used correlation analysis to point out that *L. alimentarius* was highly correlated with the formation of 10 free amino acids during the natural fermentation of pickled chayote [43]. In addition, *Enterobacter* was also observed to have a significant positive correlation with 11 free amino acids (*p* < 0.05). In Spanish fermented meat products, *Enterobacter* was isolated and, when cultured in the medium, produced large amounts of free amino acids [44]. As a filamentous fungus, *Monascus* can secrete a large number of proteases to promote the release of free amino acids [15]. Compared to unfermented soybeans, *Monascus* fermented soybeans achieved a 4.5-fold increase in free amino acids and a significant increase in γ-aminobutyric acid (GABA) [45]. *Bacillus* is the dominant bacteria in most traditional fermented soy foods, and some of them exhibit strong amylase, protease, and lipase activities [46]. Jang used strains of four *Bacillus* spp. to ferment soybeans and analyzed changes in free amino acid content, showing that *B*. *licheniformis* increased serine, threonine, and glutamate; *B*. *subtilis* increased alanine, asparagine, glycine, leucine, proline, tryptophan, and lysine; *B*. *velezensis* increased the GABA concentration to >200% of that in the control samples [47]. On the other hand, some free amino acid contents were negatively correlated with microbial genera, such as *Burkholderia-Caballeronia-Paraburkholderia* and *Phialemoniopsis*. As an important source of nitrogen for microbial growth, free amino acids are consumed due to the growth of miscellaneous bacteria.

As shown in Figure 3b, microbial genera were divided into two parts by cluster analysis due to different correlations with flavor compounds. Among them, *Lactobacillus*, *Lactiplantibacillus*, *Cutaneotrichosporon*, *Colletotrichum*, *Enterobacter*, *Haradamyces*, *Pallidocercospora*, *Klebsiella*, *Alternaria*, *Weissella*, *Monascus*, *Comamonas*, *Leuconostoc*, and *Bacillus* had positive correlations with most of the volatile flavor compounds. Among them, *Lactiplantibacillus* had significant positive correlations with ethyl acetate, acetic acid, and methyl isobutyrate (*p* < 0.01). Carbohydrate metabolism and amino acid metabolism are important pathways for the production of volatile flavor substances [11]. In heterolactic fermentation, *Lactobacillus* produces volatile compounds such as acetic acid and ethanol by consuming sugars. Organic acids and alcohols will produce esters through esterification reactions [27]. As the core flavor-contributing microbiota of traditional Chinese fermented vegetables, *Lactobacillus* was highly correlated with the formation of several key esters (benzoic acid ethyl ester, isopentyl formate, and ethyl octanoate) [48]. In the traditional brewing process of Chinese *Huangjiu*, LAB produce large amounts of organic acids that react with alcohols to produce ester aroma components [11]. On the other hand, *Lactiplantibacillus* was negatively correlated with methanol and 1-pentanol (*p* < 0.01). Methanol has been historically considered an exogenous product that leads to damage to the human nervous systems when consumed [49]. Previous studies have shown that *Lactobacillus* can reduce the levels of bad compounds in foods through complex metabolism; for example, the inoculation of *Lactobacillus*-fermented soybeans effectively reduces the levels of methanol, acetaldehyde, and hexanal [50]. *Cutaneotrichosporon* had a significant positive correlation with the formation of ethyl pentanoate (*p* < 0.05). *Burkholderia-Caballeronia-Paraburkholderia* had a significant positive correlation with ethyl pentanoate (*p* < 0.01). However, *Enterobacter* had a negative positive correlation with ethyl pentanoate (*p* < 0.01). This suggests that different microbial genera may lead to the increase or decrease in the same volatile flavor compounds during HQW fermentation.

Although some dominant microbial genera have no significant correlation with flavor compounds, their positive correlation is equally noteworthy, such as *Leuconostoc*, *Weissella*, and *Monascus*. *Monascus* is used for saccharification due to its amylase- and protease-producing properties, which contribute to the rapid formation of the flavor of HQW [15]. As shown in Figure 3b, *Monascus* had positive correlations with the formation of esters, alcohols, acids, aldehydes, and ketones. *Leuconostoc* and *Weissella* preferred the formation of esters and ketones. As a fermentation starter, *Leuconostoc* was shown to promote the formation of 2-butanone during milk fermentation [51]. Xiang found that the combined inoculation of *Weissella* *cibaria* and *Lactobacillus plantarum* significantly increased the content of organic acids (lactic acid, acetic acid), esters (butyl butyrate), alcohols (terpinen-4-ol, cineole, linalool, 4-(1-methylethyl)-cyclohexanol), and olefins (alpha-phellandrene, 1,3-p-menthadiene, myrcene, chrithmene) in traditional Sichuan Pickle [52]. During the fermentation of traditional Korean soybean paste, *Weissella* had a significant positive correlation with the formation of lactic acid and ethyl acetate [46].

## 4. Conclusions

The work presented the physicochemical properties, flavor compounds changes, and bacterial and fungal genera succession and their relationships during the fermentation of Hong Qu glutinous rice wine (HQW). Physicochemical properties were used to show the fermentation status of HWQ; the pH decreased and AAN and alcohol increased with prolonged fermentation time. The total sugar increased significantly at day 6 and then significantly decreased thereafter. Flavor compounds, especially Bitter-tasting BAAs (e.g., Arg and Trp), organic acids (e.g., lactic acid, citric acid, and succinic acid), and volatile compounds (e.g., esters and alcohol), accumulated significantly with prolonged fermentation. As for the microbial communities, *Pantoea*, *Lactiplantibacillus*, *Lactobacillus*, *Leuconostoc*, and *Weissella* predominated the bacterial genera during the fermentation, and *Monascus* was the most dominant fungal genus. By correlation analysis and multiple correction, *Lactiplantibacillus* was identified as a microbial genus due to its significant positive correlation with 4 organic acids, 15 free amino acids, and 3 volatile flavor compounds. This study contributes to the in-depth understanding of the different roles of microorganisms in the formation of flavor compounds during the fermentation of traditional HQW, and provides a theoretical reference for the industrial production of traditional HQW.

## Figures and Tables

**Table 1:** Changes in physicochemical properties during HQW fermentation.

Physicochemical Index	D0	D6	D12	D18	D24	D30
pH	5.83 ± 0.01 ^a^	3.86 ± 0.02 ^c^	3.90 ± 0.01 ^b^	3.85 ± 0.01 ^c^	3.79 ± 0.02 ^d^	3.84 ± 0.01 ^c^
Total sugar (g/L)	14.36 ± 0.17 ^b^	48.70 ± 1.20 ^a^	3.47 ± 0.17 ^c^	3.21 ± 0.80 ^c^	2.29 ± 0.15 ^cd^	1.56 ± 0.08 ^d^
Alcohol (*v*/*v*, %)	0.18 ± 0.02 ^e^	7.33 ± 1.10 ^d^	14.00 ± 0.28 ^c^	17.20 ± 0.30 ^b^	18.80 ± 0.87 ^a^	19.77 ± 0.15 ^a^
Amino acid nitrogen (g/L)	0.10 ± 0.004 ^f^	0.14 ± 0.01 ^e^	0.33 ± 0.02 ^d^	0.43 ± 0.004 ^c^	0.54 ± 0.004 ^b^	0.65 ± 0.01 ^a^

Values are presented as mean ± standard error (*n* = 3), ^a–f^ values with different letters in the same row are significantly different (*p* < 0.05) from each other.

**Table 2:** Changes in the free amino acids fraction of HQW during different fermentation stages.

Amino Acid (mg/L)	D0	D6	D12	D18	D24	D30
Umami						
Asp	6.40 ± 0.12 ^f^	20.31 ± 0.15 ^e^	79.83 ± 2.06 ^d^	108.34 ± 2.25 ^c^	142.99 ± 1.42 ^b^	161.20 ± 2.11 ^a^
Glu	31.24 ± 0.53 ^e^	26.41 ± 0.12 ^e^	92.7 ± 1.92 ^d^	323.69 ± 9.55 ^c^	370.19 ± 5.43 ^b^	392.54 ± 7.64 ^a^
TUAA	37.64	46.72	172.53	432.03	513.18	553.74
Sweet						
Ser	10.38 ± 0.16 ^e^	7.39 ± 0.09 ^f^	36.12 ± 0.93 ^d^	56.77 ± 1.04 ^c^	77.35 ± 1.29 ^b^	94.16 ± 2.64 ^a^
Gly	3.13 ± 0.07 ^c,d^	1.49 ± 0.01 ^e^	8.04 ± 0.25 ^b^	11.91 ± 0.25 ^a^	3.49 ± 0.64 ^c^	2.59 ± 0.08 ^d^
Thr	12.43 ± 0.27 ^f^	44.98 ± 0.25 ^e^	145.62 ± 3.23 ^d^	195.54 ± 3.98 ^c^	226.90 ± 3.98 ^b^	247.38 ± 5.50 ^a^
Met	7.21 ± 0.74 ^f^	15.97 ± 0.21 ^e^	22.71 ± 0.30 ^d^	53.72 ± 0.88 ^c^	81.95 ± 1.08 ^b^	85.64 ± 1.59 ^a^
Ala	1.43 ± 0.08 ^e^	3.26 ± 0.22 ^e^	7.49 ± 0.20 ^d^	26.98 ± 0.94 ^c^	67.06 ± 1.27 ^b^	75.28 ± 1.98 ^a^
Pro	31.05 ± 3.57 ^f^	113.86 ± 2.46 ^e^	228.6 ± 8.42 ^c^	247.15 ± 5.14 ^c^	274.8 ± 14.08 ^b^	306.4 ± 7.01 ^a^
TSAA	34.58	73.00	219.98	344.91	456.70	505.05
Bitter						
His	13.08 ± 0.19 ^f^	35 ± 0.34 ^e^	95.93 ± 1.57 ^d^	139.9 ± 4.04 ^c^	185.57 ± 3.97 ^b^	218.86 ± 6.10 ^a^
Arg	46 ± 0.76 ^f^	127.77 ± 0.87 ^e^	261.12 ± 5.7 ^d^	377.87 ± 7.37 ^c^	481.23 ± 8.74 ^b^	518.08 ± 12.91 ^a^
Lys	1.54 ± 0.70 ^c^	1.71 ± 0.31 ^c^	1.29 ± 0.48 ^c^	2.59 ± 0.26 ^b^	3.9 ± 0.23 ^a^	4.63 ± 0.10 ^a^
Val	4.03 ± 0.07 ^e^	3.1 ± 0.09 ^e^	26.86 ± 0.64 ^d^	50.63 ± 0.98 ^c^	73.92 ± 1.07 ^b^	91.02 ± 2.43 ^a^
Trp	50.46 ± 1.51 ^f^	132.71 ± 0.53 ^e^	475.03 ± 10.21 ^d^	613.99 ± 13.01 ^c^	769.46 ± 9.59 ^b^	871.73 ± 14.36 ^a^
Phe	4.87 ± 0.15 ^f^	12.81 ± 0.05 ^e^	45.85 ± 0.99 ^d^	59.26 ± 1.26 ^c^	74.26 ± 0.93 ^b^	84.13 ± 1.39 ^a^
Ile	10.62 ± 0.17 ^f^	36.65 ± 0.63 ^e^	154.35 ± 4.67 ^d^	216.44 ± 4.41 ^c^	277.72 ± 4.31 ^b^	318.41 ± 6.75 ^a^
Leu	4.44 ± 1.03 ^e^	3.3 ± 0.06 ^e^	27.42 ± 0.23 ^d^	38.2 ± 1.55 ^c^	48.74 ± 0.42 ^b^	57.23 ± 1.05 ^a^
TBAA	135.04	353.05	1087.85	1498.88	1914.80	2164.09
Astringent						
Tyr	16.46 ± 0.45 ^f^	38.79 ± 0.17 ^e^	89.47 ± 2.04 ^d^	113.22 ± 1.72 ^c^	130.5 ± 1.9 ^b^	140.08 ± 2.7 ^a^
TAAA	16.46	38.79	89.47	113.22	130.5	140.08
TAA	254.77	625.42	1798.43	2636.19	3289.98	3669.36

Values are presented as mean ± standard error (*n* = 3), ^a–f^ values with different letters in the same row are significantly different (*p* < 0.05) from each other.

**Table 3:** Changes in the organic acids fraction of HQW during fermentation.

Organic Acid (mg/L)	D0	D6	D12	D18	D24	D30
Oxalic acid	54.3 ± 0.1 ^c^	37.1 ± 0.8 ^d^	59.4 ± 2.6 ^b^	61.9 ± 0.9 ^a^	57.4 ± 0.5 ^b^	53.0 ± 0.1 ^c^
Tartaric acid	785.0 ± 10.5 ^d^	784.7 ± 9.1 ^d^	1102.0 ± 4.5 ^a^	1067.5 ± 18.5 ^a,b^	1014.4 ± 17.4 ^b^	953.9 ± 14.1 ^c^
Lactic acid	27.1 ± 0.6 ^f^	4027.6 ± 6.5 ^e^	6486.9 ± 12.3 ^d^	7253.0 ± 11.7 ^c^	7654.2 ± 79.0 ^b^	8073.9 ± 46.9 ^a^
Citric acid	106.7 ± 2.2 ^d^	582.2 ± 17.0 ^c,d^	650.0 ± 11.5 ^c,d^	1086.0 ± 22.5b ^c^	1559.2 ± 23.2 ^a,b^	1877.4 ± 38.0 ^a^
Succinic acid	58.9 ± 0.6 ^f^	656.4 ± 18.6 ^e^	1056.3 ± 3.3 ^d^	1347.7 ± 6.7 ^c^	1808.5 ± 63.4 ^b^	2422.4 ± 13.0 ^a^
Malic acid	128.5 ± 2.8 ^e^	104.0 ± 5.4 ^c,d^	196.3 ± 9.7 ^d^	560.9 ± 13.7 ^c^	1123.6 ± 35.5 ^b^	1223.2 ± 38.4 ^a^
Total	1160.5	6192.0	9550.9	11,377.0	13,217.3	14,603.8

Values are presented as mean ± standard error (*n* = 3), ^a–f^ values with different letters in the same row are significantly different (*p* < 0.05) from each other.

## Data Availability

Data is contained within the article.
